# Supplementary material for: The impact of malaria-protective red blood cell polymorphisms on parasite biomass in children with severe Plasmodium falciparum malaria
Source: Nat Commun. 2022 Jun 8;13:3307. doi: 10.1038/s41467-022-30990-5 (PMC9178016; doi:10.1038/s41467-022-30990-5)
Supplement: Supplementary file 1 — Supplementary Information [file 41467_2022_30990_MOESM1_ESM.docx]

Supplementary information: “The impact of malaria-protective red blood cell polymorphisms on parasite biomass in children with severe *Plasmodium falciparum* malaria”

**
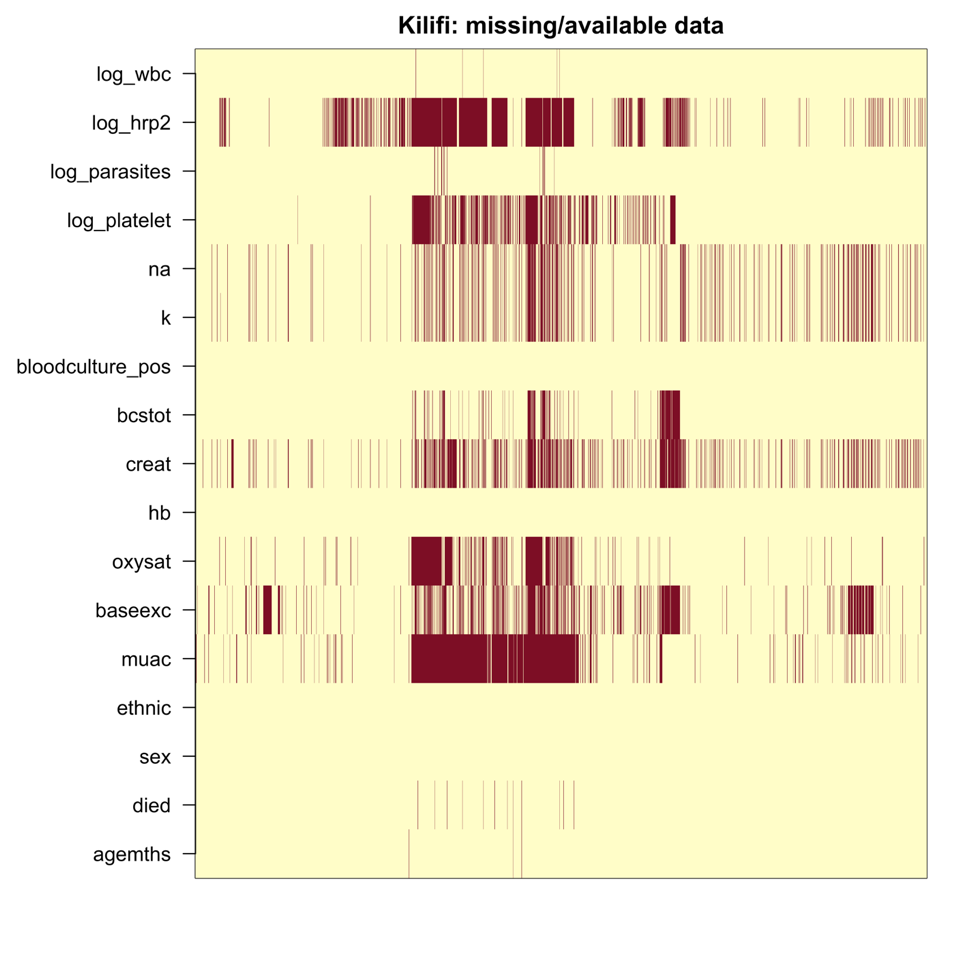
**

**Supplementary Figure 1: Pattern of missing data in the clinical dataset (red: missing; yellow: not missing).** Patients (columns) are ordered by date of enrolment.

**
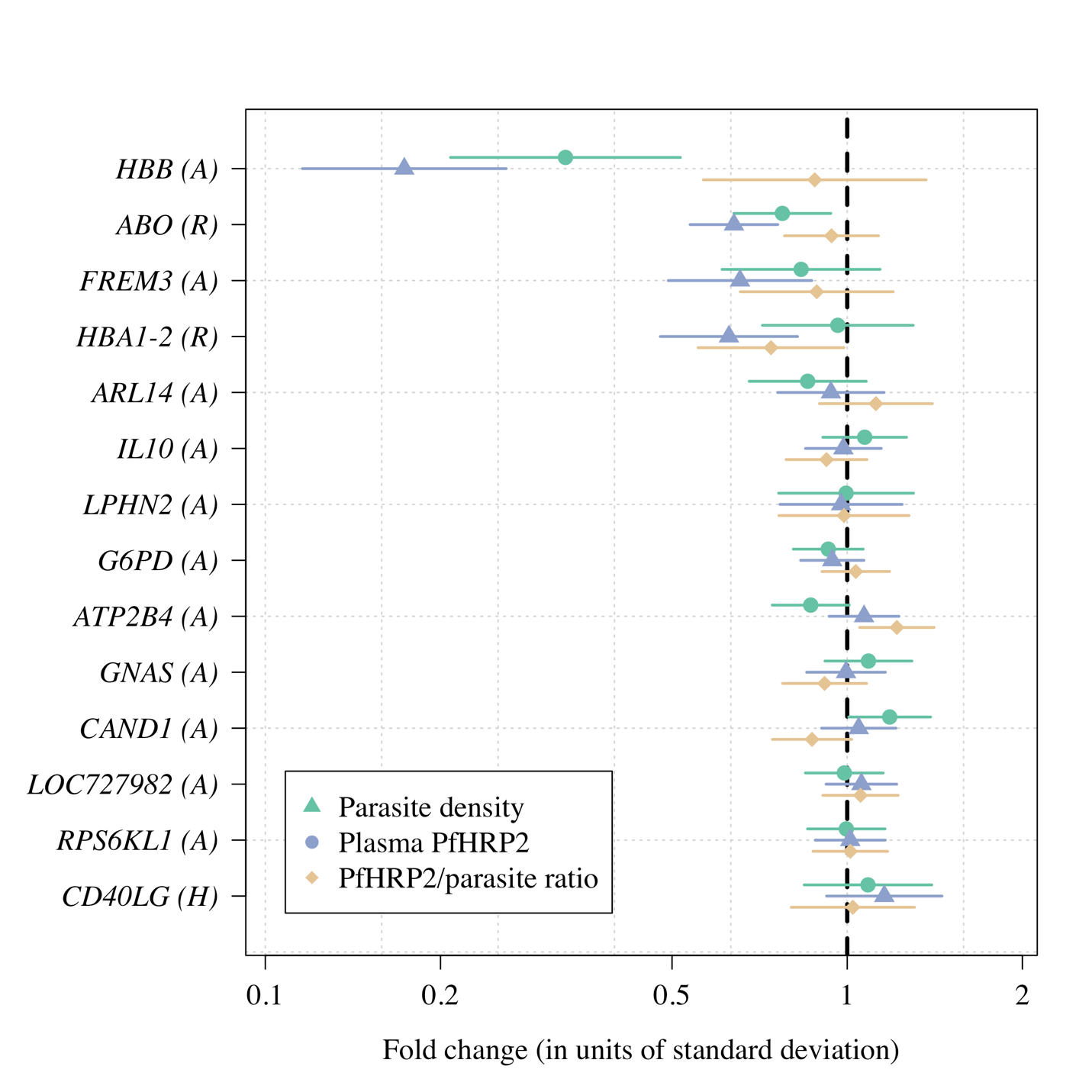
**

**Supplementary Figure 2:** **Case-only genetic association study using 14 targeted variants and three quantitative traits.** Effect estimates (95% confidence intervals) expressed as fold changes on the standardised trait distribution (rescaled to have mean 0 and standard deviation 1). This figure is the same as Figure 1 in the main text except that the linear models are include the following patient covariates: hemoglobin, age, sex, and bacteremia.

**
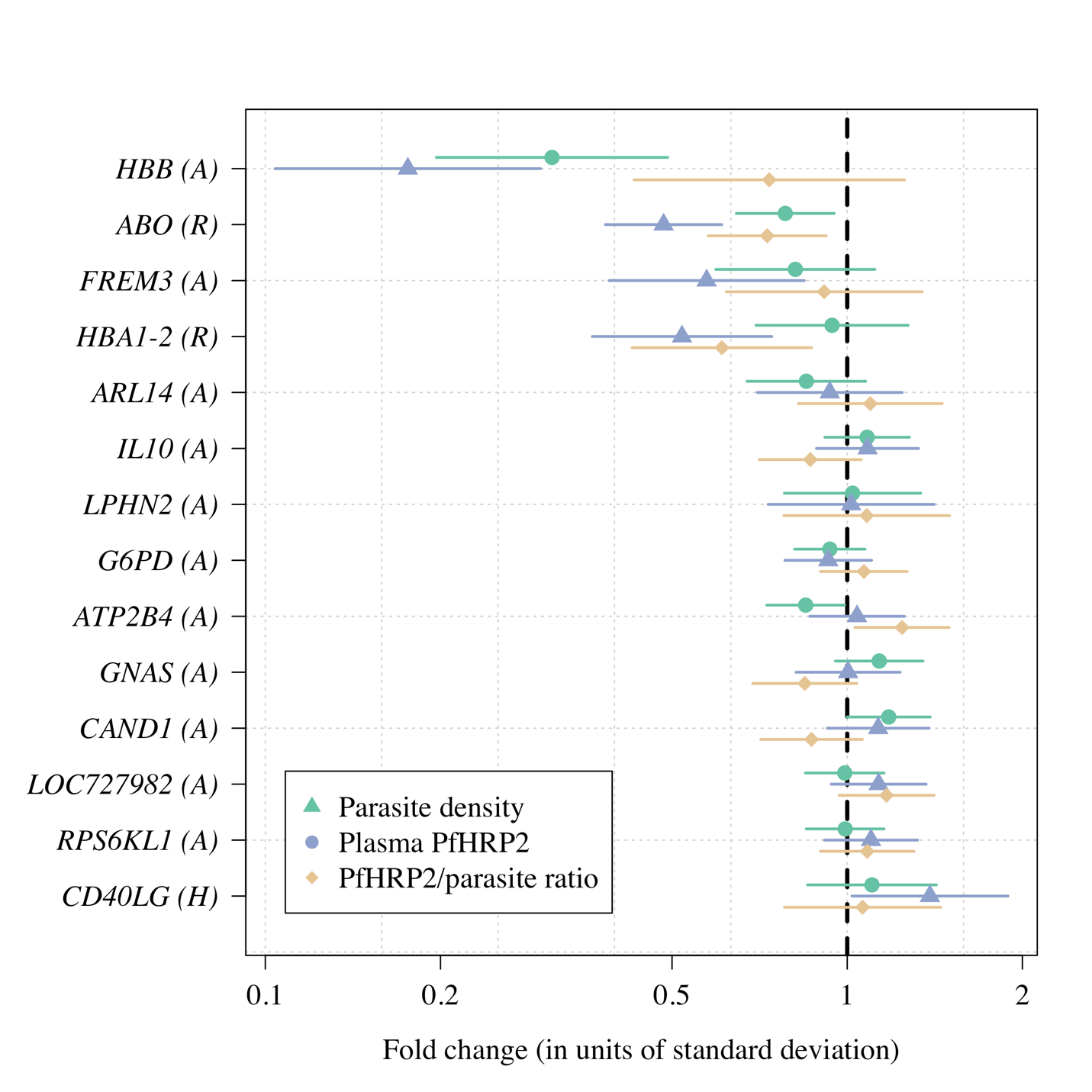
**

**Supplementary Figure 3: Case-only genetic association study using 14 targeted variants and three quantitative traits.** Effect estimates (95% confidence intervals) expressed as fold changes on the standardised trait distribution (rescaled to have mean 0 and standard deviation 1). This figure is the same as Figure 1 in the main text except that the linear models are fitted using complete case data only.

**
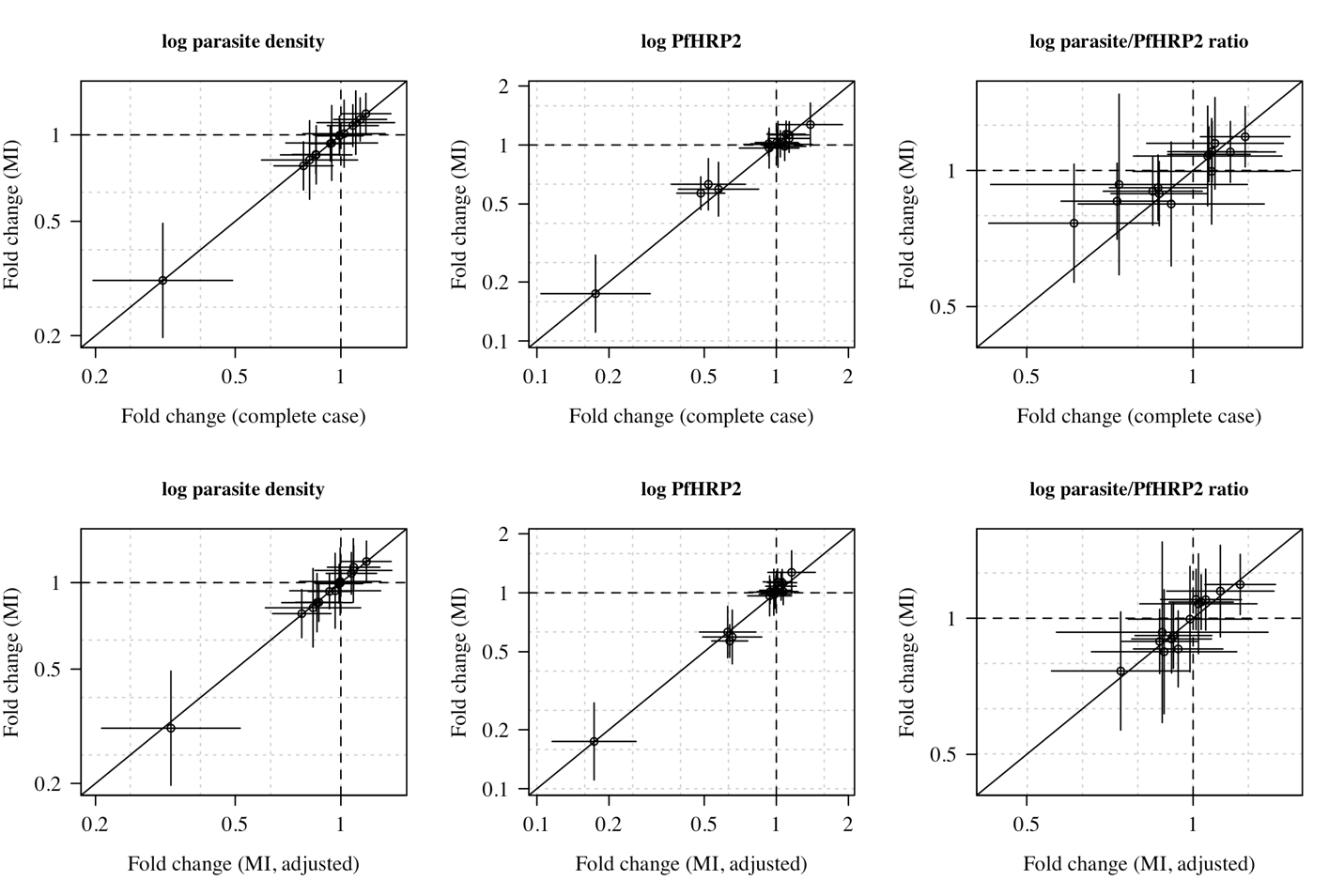
**

**Supplementary Figure 4: relationship between effect estimates for the three quantitative traits using multiple imputation (y-axis) versus complete case data only (x-axis, top row) or multiple imputation with adjustment for hemoglobin, age and bacteremia (x-axis, bottom row)**. Circles show point estimates, lines show 95% confidence intervals.

**
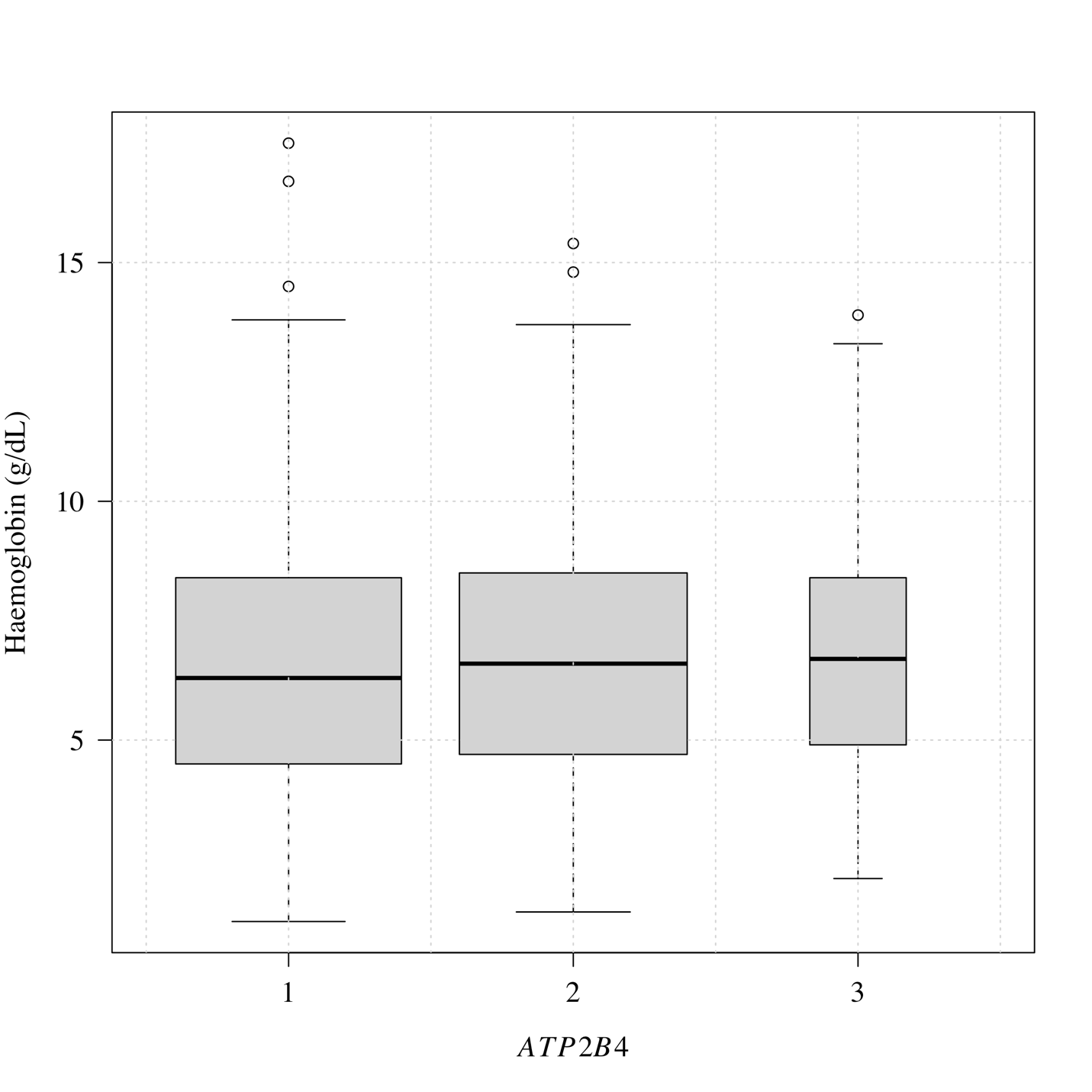
**

**Supplementary Figure 5: boxplots (center: median; bounds of box: interquartile range; whiskers: 1.5 standard deviations; points: outliers) showing the relationship between the ATP2B4 genotypes and admission hemoglobin.**

| Gene (polymorphism) | aa or a  (controls) | aA (controls) | AA or A (controls) | aa or a  (cases) | aA  (cases) | AA or A  (cases) |
| --- | --- | --- | --- | --- | --- | --- |
| *HBB*  (rs334) | 0.8 | 15.1 | 84.1 | 0.5 | 2.6 | 96.9 |
| *FREM3* (rs186873296) | 1.0 | 17.0 | 82.0 | 0.2 | 10.6 | 89.1 |
| *ABO*  (rs8176719) | 6.6 | 38.7 | 54.7 | 8.8 | 44.2 | 47.0 |
| *ATP2B4* (rs1541255) | 10.7 | 45.1 | 44.2 | 8.3 | 46.3 | 45.4 |
| *HBA1-2*  (α^-3.7^-thalassaemia) | 16.1 | 49.5 | 34.4 | 12.3 | 48.0 | 39.7 |
| *G6PD* (rs1050828) | 12.2 | 13.8 | 74.0 | 12.2 | 13.8 | 74.0 |
| *CD40LG* (rs3092945) | 13.3 | 16.4 | 70.3 | 17.1 | 17.5 | 65.4 |
| *RPS6KL1* (rs3742785) | 9.6 | 38.3 | 52.1 | 8.9 | 42.2 | 48.9 |
| *LOC727982* (rs1371478) | 8.2 | 37.5 | 54.3 | 7.5 | 43.5 | 49.0 |
| *ARL14* (rs75731597) | 1.0 | 14.4 | 84.7 | 0.8 | 18.4 | 80.7 |
| *LPHN2* (rs72933304) | 0.7 | 15.7 | 83.6 | 0.4 | 13.5 | 86.1 |
| *IL10*  (rs1800890) | 6.5 | 36.0 | 57.5 | 4.3 | 36.9 | 58.8 |
| *CAND1* (rs10459266) | 4.8 | 32.4 | 62.8 | 5.2 | 34.9 | 59.9 |
| *GNAS*  (rs8386) | 3.2 | 31.4 | 65.4 | 3.9 | 29.6 | 66.5 |

**Supplementary Table 1:** **Genotype frequencies in controls and cases**. Data are expressed as proportions (%) in the controls (n=3940) and in the cases (n=2198). The minor allele (defined by the control frequencies) is denoted a and major allele A. For X-chromosome genes (G6PD and CD40LG) the tables show aggregated frequencies of hemizygotes and homozygotes (a and aa; A and AA).

**Supplementary Table 2: Point estimates and upper & lower bounds of the 95% confidence intervals for the best fitting models for the 14 polymorphisms regressed on the parasite density.** A: additive; H: heterozygous; R: recessive.

**Supplementary Table 3: Point estimates and upper & lower bounds of the 95% confidence intervals for the best fitting models for the 14 polymorphisms regressed on the plasma PfHRP2 concentration**. A: additive; H: heterozygous; R: recessive.

**Supplementary Table 4: Point estimates and upper & lower bounds of the 95% confidence intervals for the best fitting models for the 14 polymorphisms regressed on the HRP2 to parasite ratio.** A: additive; H: heterozygous; R: recessive.
